# Two-dimensional Topological Crystalline Insulator Phase in Sb/Bi Planar Honeycomb with Tunable Dirac Gap

**DOI:** 10.1038/srep18993

**Published:** 2016-01-14

**Authors:** Chia-Hsiu Hsu, Zhi-Quan Huang, Christian P. Crisostomo, Liang-Zi Yao, Feng-Chuan Chuang, Yu-Tzu Liu, Baokai Wang, Chuang-Han Hsu, Chi-Cheng Lee, Hsin Lin, Arun Bansil

**Affiliations:** 1 Department of Physics, National Sun Yat-Sen University, Kaohsiung 804, Taiwan; 2 Centre for Advanced 2D Materials and Graphene Research Centre, National University of Singapore, Singapore 117546; 3 Department of Physics, National University of Singapore, Singapore 117542; 4 Department of Physics, Northeastern University, Boston, Massachusetts 02115, USA

## Abstract

We predict planar Sb/Bi honeycomb to harbor a two-dimensional (2D) topological crystalline insulator (TCI) phase based on first-principles computations. Although buckled Sb and Bi honeycombs support 2D topological insulator (TI) phases, their structure becomes planar under tensile strain. The planar Sb/Bi honeycomb structure restores the mirror symmetry, and is shown to exhibit non-zero mirror Chern numbers, indicating that the system can host topologically protected edge states. Our computations show that the electronic spectrum of a planar Sb/Bi nanoribbon with armchair or zigzag edges contains two Dirac cones within the band gap and an even number of edge bands crossing the Fermi level. Lattice constant of the planar Sb honeycomb is found to nearly match that of hexagonal-BN. The Sb nanoribbon on hexagonal-BN exhibits gapped edge states, which we show to be tunable by an out-of-the-plane electric field, providing controllable gating of edge state important for device applications.

Topological crystalline insulators (TCIs) are a recently discovered novel phase of quantum matter in which topological protection of states arises from a combination of time-reversal and crystalline symmetries^1^,^2^. TCIs are to be contrasted sharply from the more common topological insulators (TIs) where time-reversal symmetry (TRS) alone is the source of topological protection of states^3^,^4^,^5^,^6^. In particular, the 2D TIs, also referred to as quantum spin Hall (QSH) insulators, can support dissipationless edge currents since the only available scattering channel (backscattering) is forbidden by symmetry constraints^3^,^4^,^5^,^6^. In the panorama of topological materials, the tunable surface/edge states of the TCIs could provide unique platforms for designing next generation electronics and energy and information technologies.

SnTe class of materials was theoretically predicted to harbor the three-dimensional (3D) TCI phase^2^, and this class of material still remains the only such phase that has been realized experimentally. Properties of SnTe^7^,^8^,^9^, Pb_1−*x*_Sn_*x*_Te^10^,^11^, and Pb_1−*x*_Sn_*x*_Se^12^,^13^,^14^ have been investigated theoretically and experimentally. In a recent report, the freestanding monolayer PbSe is predicted to be 2D TCI^15^, although this has not so far been realized experimentally. On the other hand, many thin films of groups IV and V, III-V compounds^16^,^17^,^18^,^19^,^20^,^21^,^22^,^23^,^24^,^25^, and their films passivated by hydrogens or halogens^26^,^27^,^28^,^29^,^30^,^31^,^32^,^33^,^34^,^35^ are predicted to harbor the 2D-TI order in the honeycomb structure. This provides strong motivation for exploring the possibility that TCI phases could be hidden in 2D honeycombs of thin films of other materials. Strained Sb and Bi buckled honeycombs are especially germane in this connection because they assume a planar structure under strain^22^, and thus restore the mirror symmetry of the pristine honeycomb, opening up a new playground for the discovery of 2D-TCI phases.

In this study, we identify the pristine Sb/Bi honeycomb as a 2D-TCI in its planar form via first-principles calculations. With increasing in-plane strain, the buckled Sb/Bi honeycomb first undergoes a transition from a Z_2_ nontrivial to a trivial phase through a band inversion at the *M* point, before it assumes the planar structure. Further increase in strain leads to the appearance of the TCI phase in the planar Sb/Bi honeycomb, which is protected by the reflection symmetry to the planar structure. Since the corresponding Z_2_ invariant is zero, a non-zero mirror Chern number, thus supports its 2D-TCI character. We also investigated nanoribbons of Sb/Bi planar honeycomb with both armchair as well as zigzag edges, and found the presence of two Dirac cones with nodes lying within the band gap with an even number of edge bands crossing the Fermi level as signature of TCIs. Keeping in mind that a film must eventually be placed or grown on a substrate, we propose that hexagonal-BN (h-BN) is a good candidate substrate because the lattice constants of planar Sb honeycomb and h-BN are nearly commensurate. In this connection, we consider the electronic structure of a planar Sb nanoribbon on h-BN, and found that it supports two gapped Dirac cones, reflecting the substrate-induced symmetry breaking. This substrate-induced gap, however, is shown to be tunable with an out-of-the-plane electric field, providing a useful materials platform for applications.

## Results

**Figure 1:**
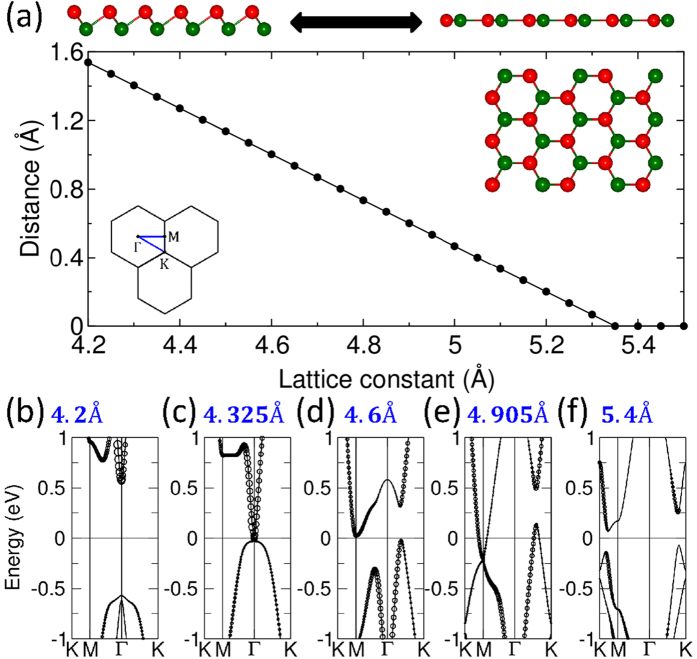
(**a**) A schematic view of the transformation of Sb/Bi buckled honeycomb into a planar honeycomb. The plot gives buckling height as a function of lattice constant for the Sb honeycomb. Top view of the buckled honeycomb structure and the associated 2D Brillouin zone and high symmetry points are also shown. The atoms of two different colors mark atoms in the two different planes of the buckled structure. (**b**–**f**) give the band structures over a wide range of lattice constants.

The structural phase transition of freestanding Sb/Bi honeycomb from buckled to planar structure under a tensile strain, as well as the crystal structure and the associated 2D Brillouin zone (BZ), are shown in Fig. 1(a). The equilibrium lattice constants of the buckled and planar Sb honeycombs are 4.12 Å and 5.04 Å^25^, respectively; while the corresponding lattice constants for Bi honeycombs are 4.33 Å (buckled) and 5.27 Å (planar)^22^. Concerning topological properties, note that previous studies based on an analysis of parities of states at high symmetry points in the BZ, have shown that planar Sb and Bi honeycombs are both Z_2_ topologically trivial^22^,^24^. Since we employ the general method of ref. 36 for computing the *Z*_2_ invariant throughout this study, we have verified that the band topologies of Sb/Bi planar honeycombs we obtain are indeed Z_2_ trivial, as is also the case for the buckled Sb honeycomb.

Topological phase transitions can be analyzed by considering the evolution of band structure as a function of the lattice constant. In the case of the Sb honeycomb, two successive band inversions are seen with increasing tensile strain in Fig. 1(b–f). [Here, the black circles are proportional to the contribution of *s* orbitals]. The first inversion, which is seen to occur at the Γ-point, is associated with the slightly larger lattice constant *a* = 4.325 Å, while the second inversion takes place at the M-point with *a* = 4.905 Å, before the formation of the planar structure. The Bi honeycombs also undergo two similar band inversions (not shown in Fig. 1 for brevity)^22^. We find that the buckled Sb honeycomb at the equilibrium lattice constant is topologically trivial (Z_2_ = 0), and that after the first band inversion, the system becomes nontrivial. Interestingly, the system returns to the trivial phase once again after the second band inversion at the M-point. In sharp contrast, the Bi honeycomb is Z_2_ nontrivial in the equilibrium state, but the inversion at the M-point under tensile strain gives rise to a trivial topological phase.

We emphasize that once the planar structure is formed, the film not only possesses the full six-fold rotational symmetry but that its mirror symmetry is also restored in that the honeycomb behaves as a mirror plane with reflection symmetry. Accordingly, we examine the associated mirror Chern numbers of the planar honeycombs. For this purpose, at each *k* point, Bloch states can be classified into two groups by their mirror eigenvalues: 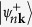 and 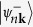, where +(−) denotes the mirror eigenvalue +*i*(−*i*). The Chern number for each mirror sector can then be computed via^37^


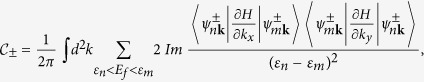


where *E*_*f*_ is the Fermi energy, and the integration is over the whole 2D BZ. In this way, we obtained mirror chern number 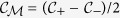 of 2 for both the Sb and Bi planar honeycombs, which indicates that these films are TCIs with an even number of edge bands crossing the Fermi level.

**Figure 2:**
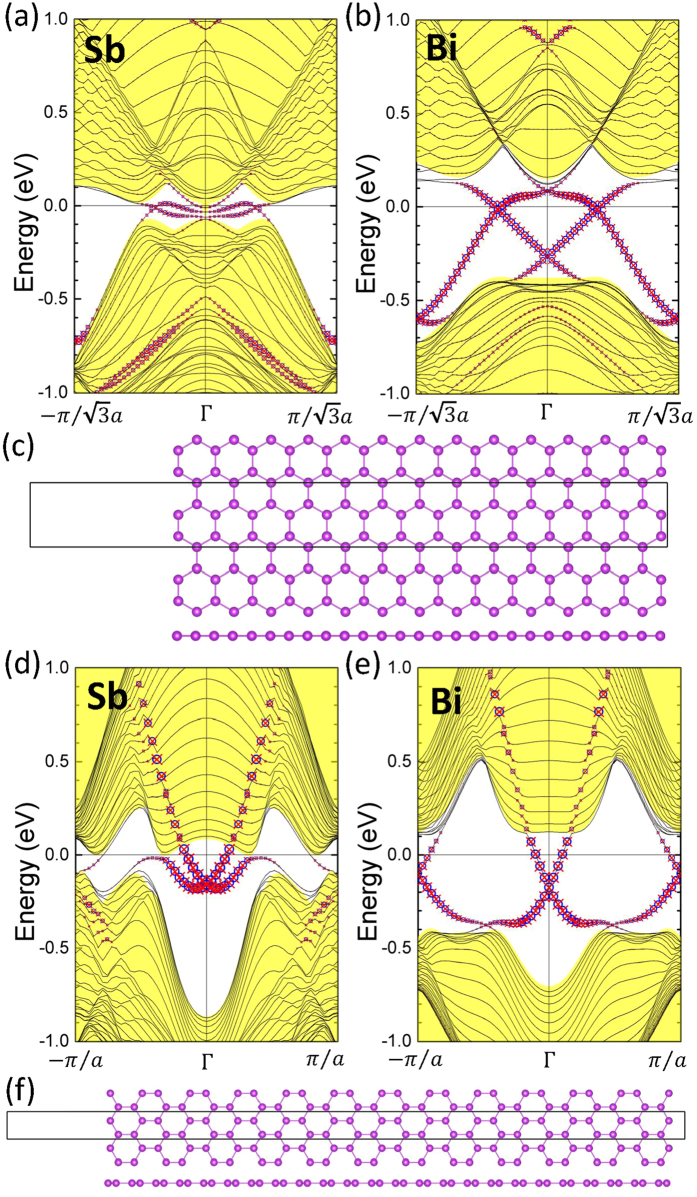
Band structures along the armchair edges of the planar Sb (**a**) and Bi (**b**) honeycombs. Structures of the nanoribbons with armchair (**c**) and zigzag (**f**) edges. Band structures for the zigzag ribbons for Sb (**d**) and Bi (**e**). Contribution of the right (left) hand side edge is marked with red crosses (blue circles). The filled yellow region denotes the bulk bands. Sizes of red crosses and blue circles are proportional to the contribution of the edges.

Insight into the nature of the protected edge states is obtained by constructing nanoribbons of planar Sb and Bi honeycombs armchair and zigzag edges, see Fig. 2(c,f). Here, instead of using the equilibrium lattice constant, we used a lattice constant of 5.229 Å, which is comparable with the lattice constant of 2 × 2 h-BN. [These results will also be helpful in comparing the edge states of Sb/Bi planar honeycomb on a 2 × 2 h-BN substrate in the discussion below]. The widths of ribbons with zigzag and armchair edges were set at 108.7 Å 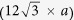 and 73.2 Å (14 × *a*), respectively. These values are large enough so that interactions between the two edges of the ribbon can be neglected. The resulting band structures are shown in Fig. 2 in which contributions of the left and right hand side edges are identified along with the bulk band structures.

The armchair as well as the zigzag edges in Fig. 2 are seen to exhibit the presence of two edge states related Dirac cones whose nodes lie within the bulk band gap. Recall that the Z_2_ topological phase can be identified by counting the number of edge bands crossing the Fermi level in half the BZ. An odd number of crossings between two time-reversal invariant momentum points in the BZ indicates a nontrivial nature of the band structure, whereas an even number of such crossings corresponds to the Z_2_ trivial order. Here, however, we see in Fig. 2, that there is an even number of edge bands cutting across the Fermi level for both Sb and Bi ribbons. This is consistent with the computed value of 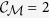, further verifying that our planar Sb and Bi honeycombs support the 2D-TCI phase.

**Figure 3:**
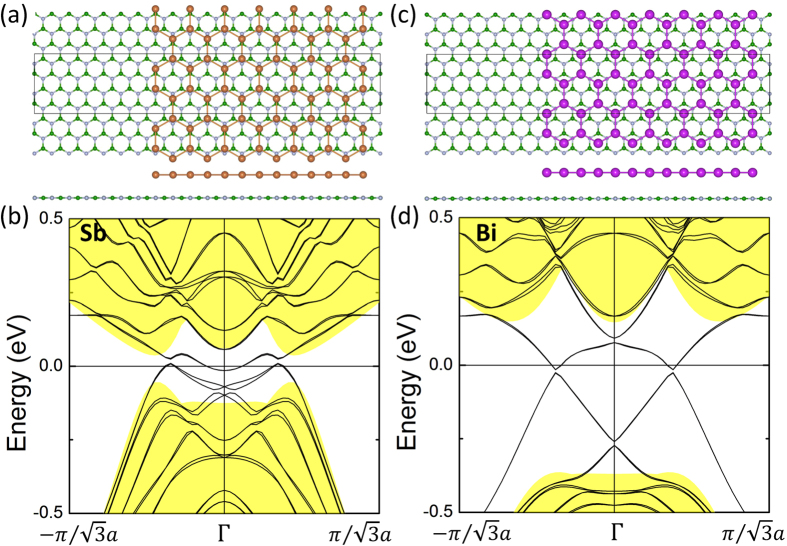
(**a**) The atomic structure and (**b**) band structure along the armchair edges of a planar Sb honeycomb on 2 × 2 h-BN. (**c**) The atomic structure and (**d**) band structure along the armchair edges of a planar Bi honeycomb on 2 × 2 h-BN. The Dirac cone opens a gap due the symmetry breaking by the h-BN substrate.

A film must eventually be placed or grown on a substrate. In this connection, we note that the 2 × 2 h-BN substrate has a lattice constant of 5.229 Å, which is quite close to the lattice constants of 5.04 Å and 5.27 Å for planar Sb and Bi honeycombs, respectively. Also, we find the total energy of the 2D-TCI films of Sb and Bi on the h-BN substrate to be insensitive to the buckling of honeycombs. [The energy difference between the planar and buckled Sb/Bi honeycomb on 2 × 2 h-BN is only 1 meV per supercell]. These considerations lead us to suggest that 2 × 2 h-BN would be a good substrate for supporting 2D-TCI films of Sb and Bi. Accordingly, we placed planar Sb and Bi ribbons of Fig. 2 on 2 × 2 h-BN substrate as shown in Fig. 3(a,c). Substrate-induced band gaps can be seen at Γ as well as in the Dirac cones lying between the Γ and M points (see Fig. 3(b,d)). Since the Dirac cone at Γ is protected by time-reversal symmetry, the gap opening at Γ reflects size effects resulting from the finite width of the ribbon. In contrast, the Dirac cone lying between Γ and M points is protected by the mirror symmetry, and for this reason, the gap opening is now also due to the breaking of the mirror symmetry in the presence of the substrate. Note that as the width of the ribbon is increased in the computations, we would expect the gap at Γ to become smaller and eventually vanish, while the gap in the Dirac cone between Γ and M will remain.

**Figure 4:**
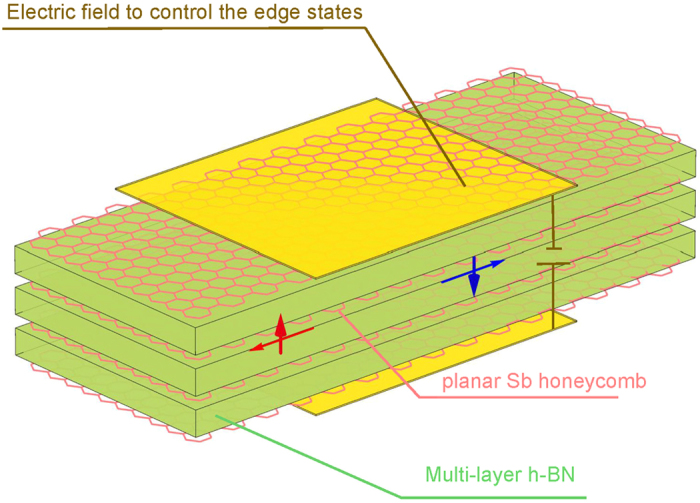
Schematic design of a proposed device composed of a Bi or Sb planar honeycomb sandwiched between h-BN substrates. The TCI edge states provide the conducting channels for spin polarized current with an out-of-the-plane electric field providing on/off control.

The sizes and nature of gaps in TIs can be tuned by an out-of-the-plane electric field^38^. This can also be expected for Dirac cones in TCIs. For example, in the case of a armchair ribbon, we have noted above that the h-BN substrate can open a gap in the Dirac cone by breaking the mirror symmetry of the film, providing a pathway for an external on/off control of edge current via gating. Keeping in mind that the h-BN substrate is a good insulator, we show schematically in Fig. 4 a proposed design of a field effect transistor based on multi-layers of h-BN and Sb/Bi planar honeycomb in which multiple TCI edge states can be used for transport.

## Conclusions

Using first-principles computations, we have explored the viability of realizing a 2D-TCI phase in films of Sb and Bi. Keeping in mind that the protection of topological states in a TCI is provided by a combination of time-reversal and crystalline symmetries and not just the time-reversal symmetry as is the case in a TI. We focus on planar Sb/Bi honeycombs in view of their mirror symmetry. Such a planar honeycomb stabilizes under tensile strain, even though the pristine Sb/Bi honeycombs assume a buckled structure, which does not possess the mirror symmetry. In order to analyze topological properties of planar Sb/Bi honeycombs, we evaluate the associated mirror Chern numbers, and find their value to be 2, indicating the presence of a TCI phase. Further insight into the nature of this TCI phase is obtained by computing the edge state spectrum of ribbons constructed from planar Sb/Bi honeycombs with armchair as well as zigzag edges, where we find two separate Dirac cones lying within the bulk band gap. Our analysis suggest that h-BN with a lattice constant nearly commensurate with that of Sb/Bi planar honeycombs would be a suitable substrate for maintaining the 2D-TCI phase. Our study predicts that planar Sb/Bi honeycombs harbor a 2D-TCI phase with gaps controllable with an external out-of-the-plane electric field, and throws open the possibility of using 2D-TCIs as novel applications platforms.

## Methods

First-principles calculations were carried out within the generalized gradient approximation (GGA) to the density-functional theory (DFT)^39^,^40^ using the projector-augmented-wave (PAW) method^41^ as implemented in the Vienna Ab-Initio Simulation Package (VASP)^42^,^43^. The kinetic energy cutoff was set at 400 eV and atomic positions were relaxed until the residual forces were less than 10^−3^ eV/Å. The convergence criteria for self-consistent iterations was set at 10^−6^ eV for electronic structure calculations with or without spin-orbit coupling (SOC). In order to simulate the buckled or planar honeycombs, a vacuum of at least 20 Å was included in the out-of-plane (*z*) direction, and a Γ-centered 30 × 30 × 1 Monkhorst-Pack grid^44^ was used to sample the 2D Brillouin zone. In the case of the nanoribbons, a vacuum of at least 20 Å along both the *y* and *z* directions was used, and 12 × 1 × 1 and 30 × 1 × 1 grids were used for the armchair and zigzag ribbons, respectively.

## Additional Information

**How to cite this article**: Hsu, C.-H. *et al.* Two-dimensional Topological Crystalline Insulator Phase in Sb/Bi Planar Honeycomb with Tunable Dirac Gap. *Sci. Rep.* **6**, 18993; doi: 10.1038/srep18993 (2016).
